# Optimal length of R–R interval segment window for Lorenz plot detection of paroxysmal atrial fibrillation by machine learning

**DOI:** 10.1186/s12938-020-00795-y

**Published:** 2020-06-16

**Authors:** Masaya Kisohara, Yuto Masuda, Emi Yuda, Norihiro Ueda, Junichiro Hayano

**Affiliations:** 1 Department of Medical Education, Nagoya City University Graduate School of Medical Sciences, 1 Kawasumi Mizuho-cho Mizuho-ku, Nagoya, 467-8601 Japan; 2 Kenz Division, Suzuken Co. Ltd, Suzuken Tomei Building, Himewaka-cho 6, Meito-ku, Nagoya, 4650045 Japan; 3 Department of Electrical Engineering, Tohoku University Graduate School of Engineering, Aoba 6-6-05 Aramaki, Aoba-ku, Sendai, 980-8759 Japan

**Keywords:** Artificial intelligence, Atrial fibrillation, Convolutional neural network, Holter electrocardiogram, Lorenz plot, Machine learning, Paroxysmal atrial fibrillation

## Abstract

**Background:**

Heartbeat interval Lorenz plot (LP) imaging is a promising method for detecting atrial fibrillation (AF) in long-term monitoring, but the optimal segment window length for the LP images is unknown. We examined the performance of AF detection by LP images with different segment window lengths by machine learning with convolutional neural network (CNN). LP images with a 32 × 32-pixel resolution of non-overlapping segments with lengths between 10 and 500 beats were created from R–R intervals of 24-h ECG in 52 patients with chronic AF and 58 non-AF controls as training data and in 53 patients with paroxysmal AF and 52 non-AF controls as test data. For each segment window length, discriminant models were made by fivefold cross-validation subsets of the training data and its classification performance was examined with the test data.

**Results:**

In machine learning with the training data, the averages of cross-validation scores were 0.995 and 0.999 for 10 and 20-beat LP images, respectively, and > 0.999 for 50 to 500-beat images. The classification of test data showed good performance for all segment window lengths with an accuracy from 0.970 to 0.988. Positive likelihood ratio for detecting AF segments, however, showed a convex parabolic curve linear relationship to log segment window length and peaked at 85 beats, while negative likelihood ratio showed monotonous increase with increasing segment window length.

**Conclusions:**

This study suggests that the optimal segment window length that maximizes the positive likelihood ratio for detecting paroxysmal AF with 32 × 32-pixel LP image is 85 beats.

## Background

Atrial fibrillation (AF) is the most common sustained arrhythmia, which is characterized by an irregular sequence of beat intervals with loss of physiological pacemaker function of the sinus node. AF is a major cause of ischemic stroke due to the intracardiac thrombus formation through blood stagnation in the atrium without effective contraction [1]. AF often causes embolization in the main trunk of the cerebral arteries, leading to large areas of infarction with generally poor prognosis [2]. Patients with AF can prevent serious stroke by taking oral anticoagulants suppressing thrombus formation [3]. Thus, early detection of AF could be highly beneficial for improving the prognosis. AF is classified into continuous AF (CAF) that appears persistently or permanently, and paroxysmal AF (PAF) that appears transiently [4]. Although the anticoagulant therapy is required for both CAF and PAF [3], PAF is currently under-diagnosed since it can be asymptomatic. Long-term ECG monitoring is used to diagnose PAF and, theoretically, the longer the monitoring, the higher the probability of detecting PAF and the accuracy to estimate stroke risk [5]. Therefore, it is desirable to develop an automatic AF detection system based on signals obtained from wearable devices that can be used in daily life.

**Fig. 1:**
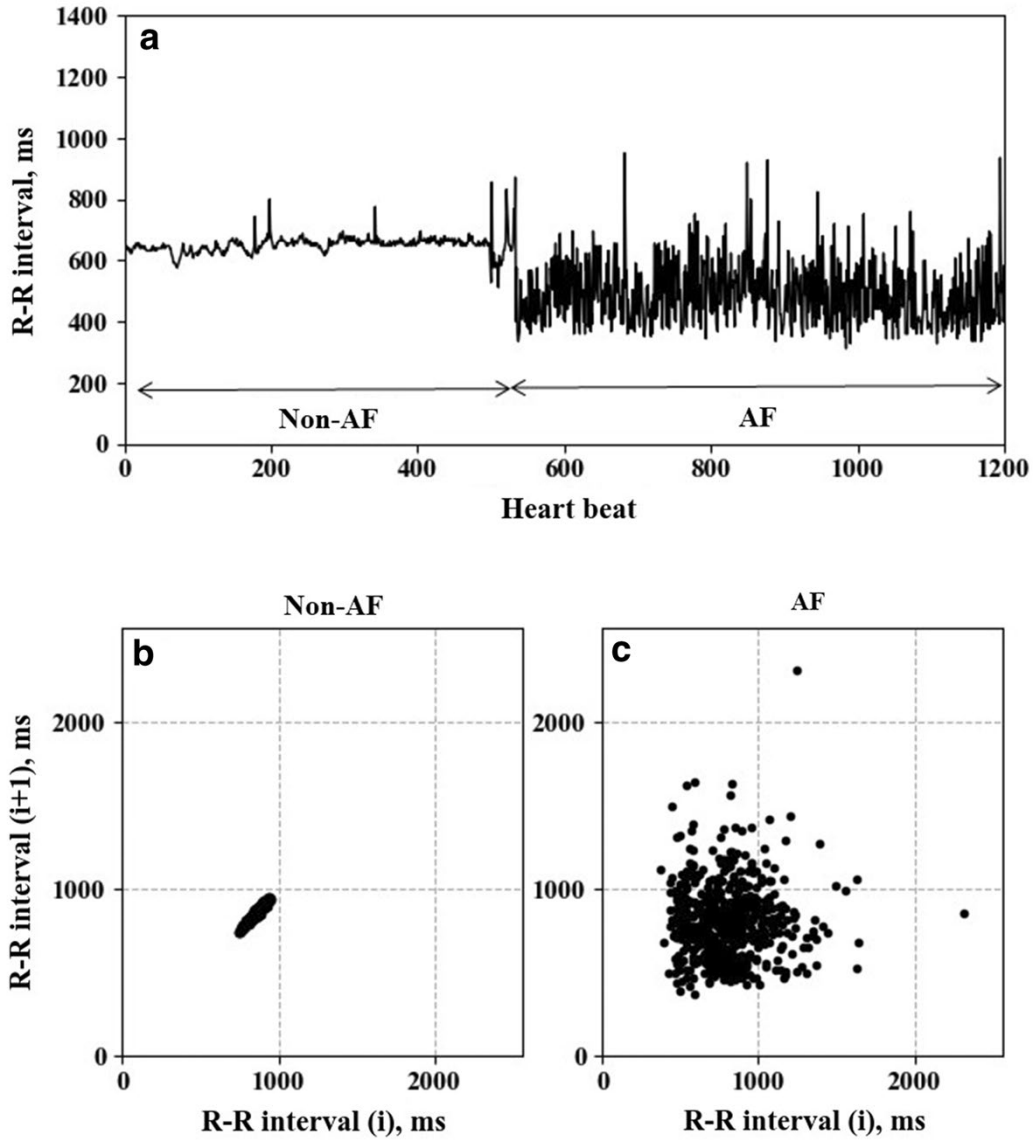
Trend graph of R–R interval time series showing a transition from non-atrial fibrillation (non-AF) to atrial fibrillation (AF) in a patient with paroxysmal AF (**a**). **b**, **c** show Lorenz plots (LP) during non-AF and AF, respectively

**Fig. 2:**
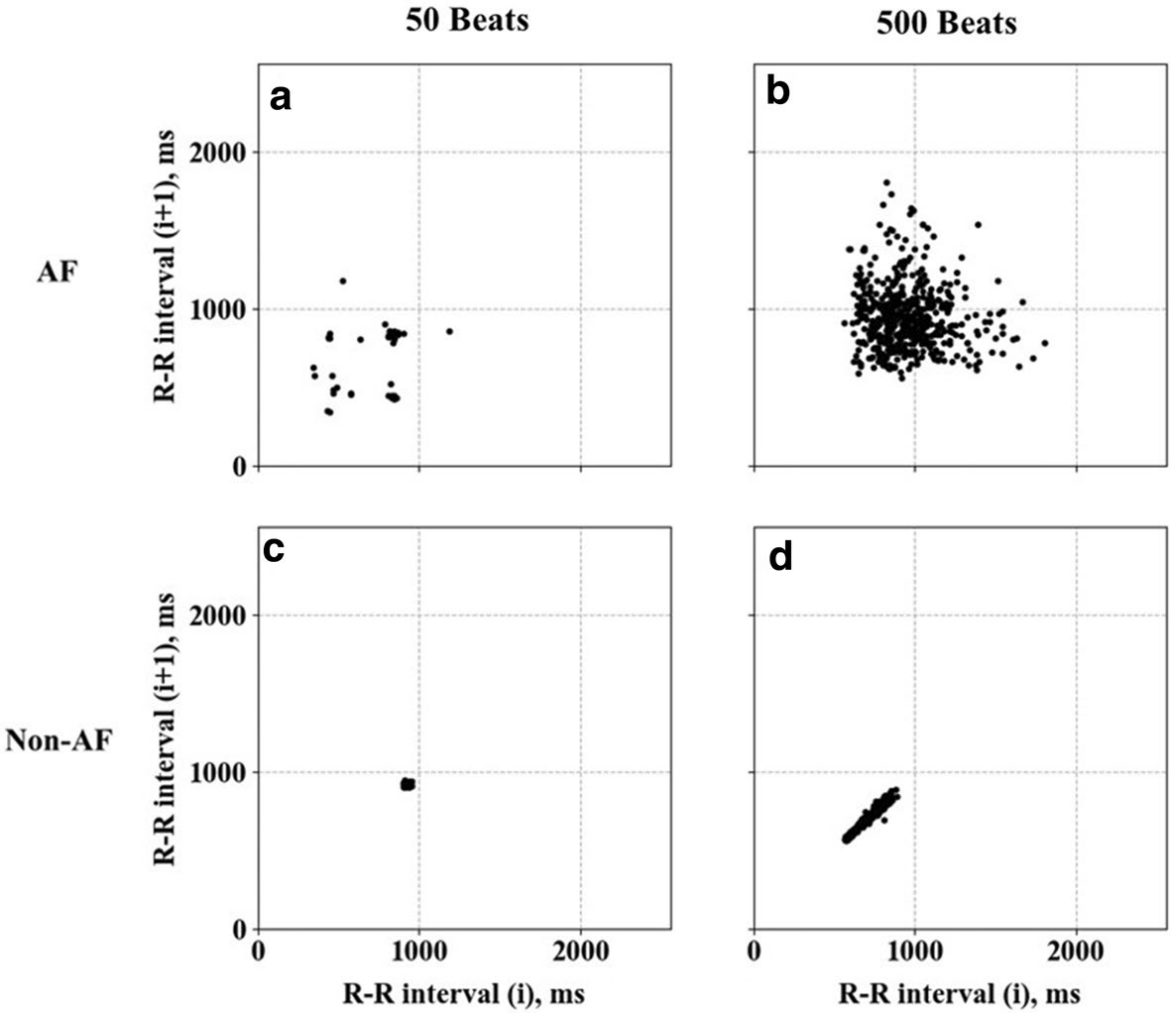
LPs of AF (**a**, **b**) and non-AF (**c**, **d**) segments with window lengths of 50 (**a**, **c**) and 500 (**b**, **d**) beats

Several automatic AF detection algorithms have been reported, and they are mostly based on features extracted from the R–R interval time-series [6–12]. Such algorithms are more robust to ambulatory noise than p-wave-based algorithms [13] and they may also be applicable to pulse interval time-series obtained from the widespread wearable pulse wave sensors. As a method to distinguish AF from other non-AF rhythms with heartbeat interval data, Lorenz plot (LP) imaging [14] has been proposed [15–18]. LP images are generated on the *X*–*Y* plane by plotting all the heartbeats in the observation segment window with the preceding interval as the *x* value and the subsequent interval as the *y* value [15, 17]. In sinus rhythm, since the difference between adjacent heartbeat intervals is small, LP shows a rod-shaped to narrow fan-shaped morphology on the diagonal line (Fig. 1). In AF, on the other hand, the plot shows a broad fan shape morphology, as the heartbeat intervals fluctuate semi-randomly without depending on the preceding interval. However, the LP-based AF detection has at least two challenges. The first is the difficulty of distinguishing AF from other arrhythmias that may exhibit irregular heartbeat intervals and when AF shows no or less irregular heartbeats such as in cases of the implanted pacemaker. In the clinical setting, these may not be a critical drawback, as detailed ECG waveform analysis is required for a definite diagnosis of arrhythmias detected by beat interval methods and of pacemaker functions. The second is the PAF detection temporal resolution, which is determined by the segment window length. Temporal resolution is important because it can affect the detection performance of short PAF and the estimation accuracy of AF burden [5]. The longer the data segment for LP, the more accurate the distinction between AF and non-AF (Fig. 2), but the lower the temporal resolution. Since no study to date has reported the optimal segment window length of LP for PAF detection, we examined the effect of segment window length of LP image on the PAF detection performance.

Recently, machine learning has become popular in medical fields [19]. For LP-based AF detection, we used a convolutional neural network (CNN) suitable for image discrimination. For different segment window lengths between 10 and 500 beats, we generated a CNN model to discriminate the LP images of AF from those of non-AF. We dared to use asymmetric datasets for training and test data; the training data were consisting of CAF and pure sinus rhythm (SR) with minimal premature beats, while the test data were consisting of PAF and non-AF including premature beats. If PAF data were used for training, LP images with a mix of AF and non-AF beats (*mixed* LP images) could be produced. Then we need to annotate such mixed LP images using certain operational criteria (for example, LP including > 50% of AF beats is annotated as AF, the other is annotated as non-AF). The CNN models generated by this method, however, may only fit the used operational criteria, and so may the obtained optimal segment window length. To avoid this, we used only CAF and pure SR for the training data so that only *pure* LP images consisting of pure AF and those of pure SR are produced. Then, we observed the behavior of the discriminant models thus generated against the test data composed of mixed LP images containing AF beats at various ratios between 0 and 100%. In this study, we used the Allostatic State Mapping by Ambulatory ECG Repository (ALLSTAR) database of 24-h ambulatory ECG [20, 21] for the training and test data and used the PhysioNet database [22] for another test data for comparisons with earlier studies.

## Results

### Characteristics of data

The characteristics of the subjects contributed to the training and test data in the ALLSTAR database are shown in Table 1. CAF cases used for the training data was older and had more frequent ventricular premature beats than PAF cases used for the test data. In PAF cases, the median frequency (IQR) of PAF episode was 4 (1-14)/24 h (range, 1-959/24 h), the median length (IOR) of individual PAF episode was 7 (4–23) beat (range, 1–115,789 beats), and the median (IQR) duration (burden) of PAF was 139.9 (10.3–579.3) min/24 h.

**Table 1:** Characteristics of subjects selected for the training and test data from the ALLSTAR database

	Training data		Test data	
	Non-AF	CAF	Non-AF	PAF
*n*	58	52	52	53
Male, %	36.2	61.5	42.3	62.3
Age, years	61.4 ± 12.5	78.0 ± 8.4	65.7 ± 11.8	68.2 ± 9.5
PAF frequency/24 h	–	–	–	4 (1–14)
Individual PAF length, beat	–	–	–	7 (4–23)
Total PAF duration, min/24 h	–	–	0	139.9 (10.3–579.3)
Total PAF beat/24 h	–	–	0	12,666 (1263–51,983)
APC/24 h	31 (10–61)	–	54 (11–150)	316 (88–1849)
VPC/24 h	5 (1–39)	48 (9–196)	10 (1–24)	6 (1–28)
24-h mean RRI, ms	844 ± 105	789 ± 141	873 ± 135	801 ± 119
24-h SD of RRI, ms	138 ± 36	217 ± 57	134 ± 43	185 ± 57

Data are frequency (%), median (IQR), or mean ± SD

*AF* atrial fibrillation, *CAF* chronic AF, *PAF* paroxysmal AF, *RRI* R–R interval, *SR* sinus rhythm, *APC* atrial premature contraction, *VPC* ventricular premature contraction

Table 2 shows the number of LP images in the training and test data from the ALLSTAR database. Of the LP images in the training data, 51% and 49% of images were annotated as non-AF and AF, respectively. The LP images in the test data were annotated by the strict criteria (annotating LP images as AF if they contained at least one AF beat, and non-AF otherwise) and 84–86% of them were annotated as non-AF, while the rest were annotated as AF. The proportion of mixed LP images increased with the segment window length.

**Table 2:** Number LP images of training and test data from ALLSTAR database

Segment window length, beat	LP images of training data, n (%)		LP images of test data, n (%)^a^		
	Non-AF	AF	Non-AF	AF (pure)^b^	AF (mixed)^c^
10	590,602 (50.7)	574,014 (49.3)	934,803 (85.5)	156,295 (14.3)	2543 (0.2)
20	295,284 (50.7)	286,996 (49.3)	467,009 (85.4)	77,426 (14.2)	2365 (0.4)
50	118,099 (50.7)	114,781 (49.3)	186,432 (85.2)	30,265 (13.8)	2000 (0.9)
100	59,035 (50.7)	57,373 (49.3)	92,967 (85.0)	14,684 (13.4)	1677 (1.5)
200	29,506 (50.7)	28,670 (49.3)	46,268 (84.7)	7033 (12.9)	1331 (2.4)
500	11,783 (50.7)	11,453 (49.3)	18,314 (83.9)	2633 (12.1)	877 (4.0)

*LP* Lorenz plot

^a^LP images were annotated with the strict criteria (LP images were annotated as AF if they contained at least one AF beat, and non-AF otherwise)

^b^LP images consisting of only AF beats (including premature beats)

^c^LP images composed of both SR and AF beats (including premature beats)

Of the LP images obtained from the PhysioNet database, 19% of images were annotated as AF by the non-strict criteria (annotating LP images as AF if they contained AF beats > 1/2 of the total beats in the segment, and non-AF otherwise).

### Machine learning with training data

The machine learning of the training data took the maximum epoch of 204 on the training of the 500-beat LP images and the minimum epoch of 96 on the 50-beat LP images. The shorter the segment window length, the longer the training time; it took 13,342 s (3.71 h) for LP images with a length of 10 beats. The cross-validation scores were 0.995 and 0.999 for 10 and 20-beat LP images, respectively, and > 0.999 for 50 to 500-beat images. The confusion matrix in the training data for each segment window length is shown in Table 3.

**Table 3:** Confusion matrix for each segment window length of the test data from ALLSTAR database

Segment window length, beats	Classification	Ground truth					
		Training data			Test data		
		Non-AF^a^	AF^b^	Accuracy	Non-AF^c^	AFc	Accuracy
10	Non-AF	588,036	3341	0.99493	903,219	930	0.970
	AF	2566	570,673		31,584	158,073	
20	Non-AF	294,986	329	0.99892	458,259	692	0.983
	AF	298	286,667		8750	79,173	
50	Non-AF	118,089	11	0.99991	184,063	671	0.986
	AF	10	114,770		2369	31,620	
100	Non-AF	59,032	2	0.99,996	92,160	527	0.988
	AF	3	57,371		807	15,838	
200	Non-AF	29,505	0	0.99998	45,759	423	0.983
	AF	1	28,670		509	7944	
500	Non-AF	11,783	0	1.00000	18,006	281	0.973
	AF	0	11,453		308	3230	

*AF* atrial fibrillation, *LP* Lorenz plot

^a^LP images obtained from pure (> 99%) SR cases

^b^LP images obtained from pure (> 99%) CAF cases

^c^Annotations of LP images with the strict criteria

### Classification performance of test data from ALLSTAR database

**Fig. 3:**
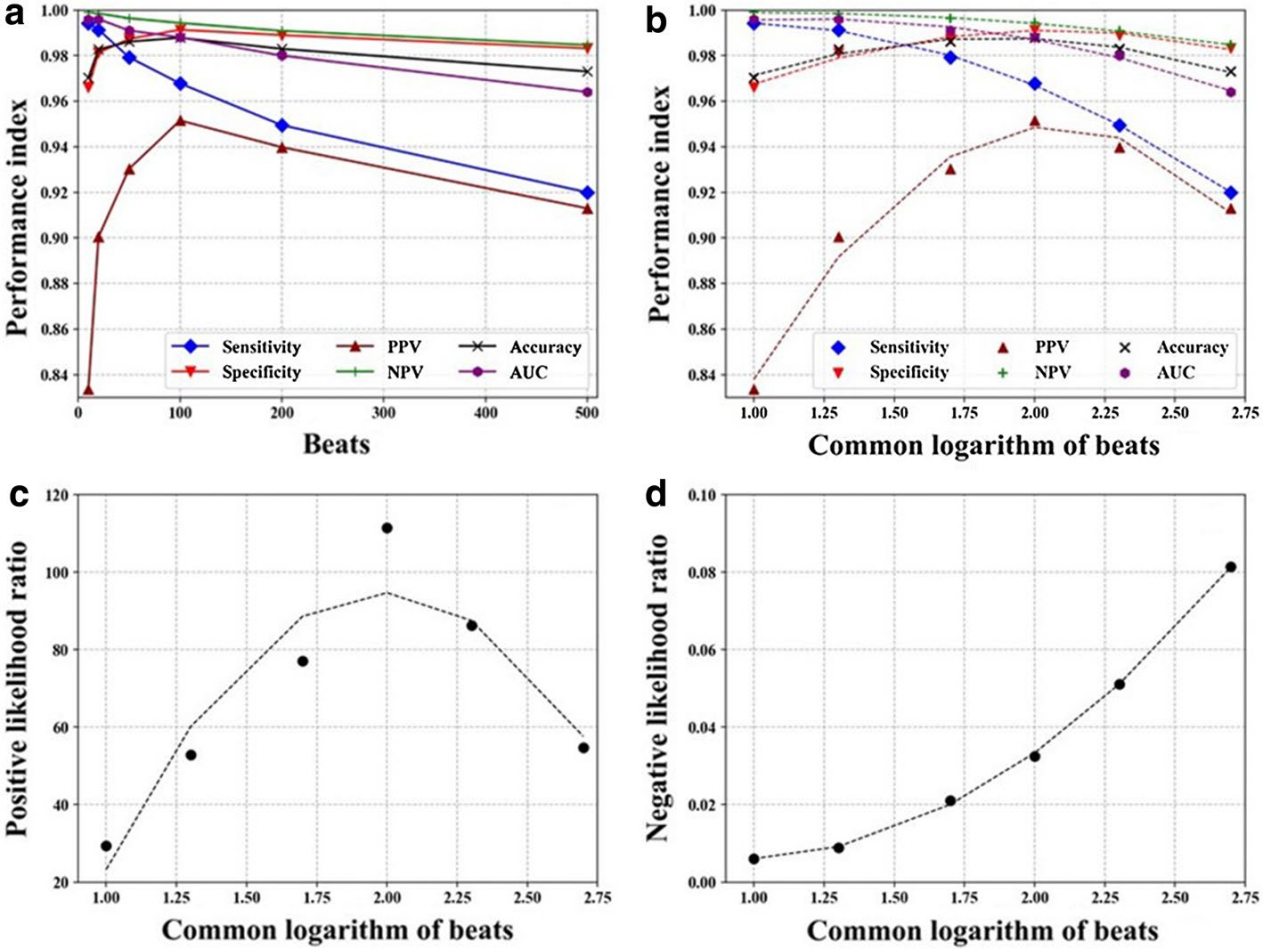
Relationships between classification performance metrics and LP segment window length in the test data from the ALLSTAR database. The LP images were annotated with the strict criteria. Panels** a** and** b** show sensitivity, specificity, positive and negative predictive values (PPV and NPV), accuracy, and area under the curve (AUC). Panels** c** and** d** show positive and negative likely hood ratios, respectively. The *x*-axis in **b**–**d** is the common log values of segment window length in beat. Dotted lines represent regression lines with parabolic functions

Table 3 also shows the confusion matrix of the test data for each segment window length. The AUC was high for all segment window lengths and the ROC curves were mostly overlapped. Figure 3a, b shows the relationships between classification performance metrics and segment window length. A maximum accuracy of 0.988 and a minimum accuracy of 0.970 were obtained at 100 and 10 beats, respectively. Sensitivity, negative-predictive value, and AUC of ROC curve declined monotonously with increasing segment window length, whereas specificity, positive-predictive value, and accuracy showed a convex parabolic relationship with log segment window length, with a peak at a segment window length of 100 beats (common log value of 2.0). At this point, sensitivity and specificity were 0.968 and 0.991, respectively. Positive likelihood ratio also showed a convex parabolic relationship with log segment window length with a peak value of 111 at a segment window length of 100 beats (Fig. 3c), whereas negative likelihood ratio increased monotonously with increasing segment window length (Fig. 3d). It showed 0.032 at a segment window length of 100 beats.

To examine the behavior of the generated CNN models against mixed LP images containing AF beats in varying proportions, the number (and proportion) of AF beats required to detect AF was analyzed in the test data from the ALLSTAR database (Table 4). The required proportions of AF beats were lower for segment window lengths of 10 and 500 beats than for 20–100 beats.

**Table 4:** Number of AF beats in LP required to detect AF in the test data from the ALLSTAR database

Segment window length, beat	Number of AF beats (%) in LP required to detect AF with given probabilities (*P*)		
	*P* = 0.50*	*P* = 0.75*	*P* = 0.95*
10	2 (20%)	3 (30%)	6 (60%)
20	7 (35%)	11 (55%)	14 (70%)
50	27 (54%)	31 (62%)	39 (79%)
100	46 (46%)	54 (54%)	65 (65%)
200	68 (34%)	95 (48%)	133 (67%)
500	90 (18%)	134 (27%)	165 (33%)

Probability of detecting AF images among all AF images annotated with the strict criteria

We also examined the model behavior against LP image containing premature beats. Table 5 shows the impact of the proportion of premature beat in LP images on the classification accuracy in the test data. For all levels of premature beat proportion, the accuracy was highest when the segment window length was 100 beats.

**Table 5:** Effects of premature beats on classification accuracy between AF and non-AF LP images

Segment window length, beat	Ratio of premature beats in LP image							
	0%	0–5%	5–10%	10–15%	15–20%	20–25%	25–30%	≥ 30%
10	0.988	–	0.869	–	0.674	–	0.495	0.388
20	0.994	0.955	0.926	0.886	0.809	0.725	0.704	0.694
50	0.996	0.972	0.936	0.900	0.861	0.791	0.748	0.822
100	0.997	0.975	0.936	0.926	0.896	0.840	0.809	0.836
200	0.997	0.974	0.919	0.919	0.806	0.688	0.773	0.768
500	0.997	0.969	0.912	0.819	0.668	0.485	0.648	0.703

Data are classification accuracy between AF and non-AF images annotated with the strict criteria

Additionally, we examined if the model shows different behavior between CAF and PAF when LP images containing solely AF beats. We produced LP from consecutive AF beats during CAF in the training data and during PAF episodes in the test data. The sensitivity for detecting AF did not differ between CAF and PAF, showing almost 100% sensitivities for both at any segment window lengths (Table 6).

**Table 6:** AF detection sensitivity for LP images consisting of > 99% AF beats during CAF and PAF episodes

Segment window length, beats	CAF	PAF episode
10	0.994	0.996
20	0.999	0.998
50	> 0.999	0.999
100	> 0.999	> 0.999
200	> 0.999	0.999
500	> 0.999	0.999

### Detailed search for optimal segment window length near 100 beats

**Fig. 4:**
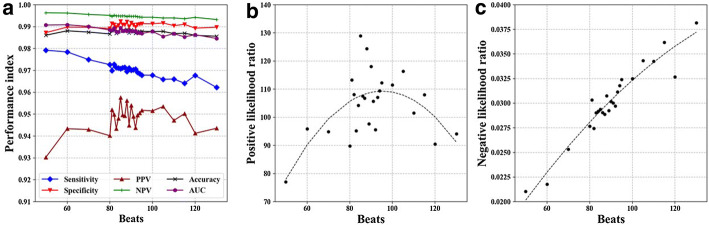
Relationships between classification performance metrics and LP segment window length with fine steps in the test data from the ALLSTAR database. The LP images were annotated with the strict criteria. *NPV* negative-predictive value, *PPV* positive-predictive value

Since the overall AF detection performance of LP image CNN model peaked at a segment window length of 100 beats, we searched for the peak performance at finer steps in length. We searched between 50 and 130 beats in 10-beat steps, especially between 80 and 95 beats in 1-beat steps. As shown in Fig. 4, the positive-predictive value and positive likelihood ratio showed variations near the peak. Regression analyses using a parabolic function estimated that the peak was at a segment window length of 85 beats.

### Estimation of AF burden

**Fig. 5:**
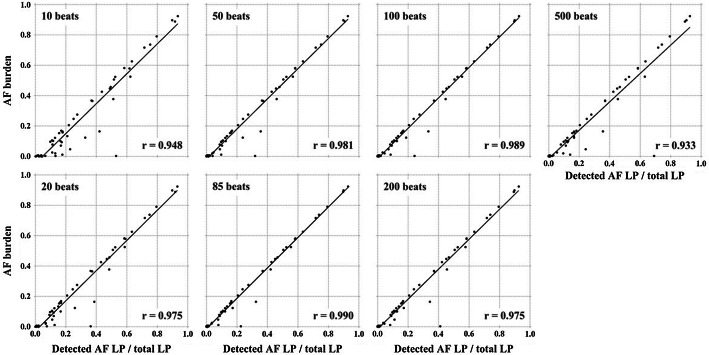
Relationship between AF burden and the ratio of detected AF LP over total LP. The number of beats written as inset of each panel represents the segment window length for LP. In each panel, each dot represents individual subject and a straight line shows the linear regression line. All correlation coefficients (*r*) are statistically significant

Figure 5 shows the relationships between AF burden and the ratio of LP detected as AF in 53 cases of the test data from the ALLSTAR database. The correlation coefficient reached a peak value of 0.99 when the segment window length was 85 beats. The agreement assessed by Bland and Altman method showed a mean difference of 0.01 and upper and lower limits of agreement of 0.03 and -0.02, respectively.

### Classification performance of test data from PhysioNet database

Table 7 shows the classification performance of the generated CNN models for LP images produced from the PhysioNet database. In these analyses, LP images was annotated by the non-strict criteria. Consistent with the results for the test data from the ALLSTAR database, a peak positive likelihood ratio was observed at the segment window length of 85 beats, where the classification accuracy was 0.979 and the AUC of ROC curve was 0.987. Table 7 also shows the performance of AF detection reported by earlier studies that used the PhysioNet database [9, 11, 13]. The classification performance of the CNN models developed in the present study was comparable to or slightly better than those of the earlier studies.

**Table 7:** Comparisons of AF detection performance in the PhysioNet database

AF detection method	Segment window length, beats	Classification performance							
		Sensitivity	Specificity	PPV	NPV	Accuracy	PLR	NLR	AUC
LP image CNN (this study)^a^	10	0.975	0.928	0.741	0.994	0.936	13.55	0.027	0.987
	20	0.980	0.958	0.830	0.996	0.962	23.24	0.021	0.989
	50	0.975	0.971	0.877	0.995	0.972	33.71	0.025	0.983
	85	0.984	0.978	0.905	0.997	0.979	45.49	0.016	0.987
	100	0.984	0.978	0.905	0.997	0.979	45.19	0.016	0.986
	200	0.992	0.976	0.897	0.998	0.979	41.08	0.008	0.982
	500	0.998	0.963	0.85	1.000	0.969	26.94	0.002	0.980
RdR map [9]	32	0.944	0.926	–	–	–	–	–	0.978
	64	0.958	0.943	–	–	–	–	–	0.986
	128	0.959	0.954	–	–	–	–	–	0.989
CoSen [13]	41	–	–	–	–	0.911	–	–	–
Poincare plot [13]	82	–	–	–	–	0.912	–	–	–
SVM [13]	65	–	–	–	–	0.909	–	–	–
Normalized fuzzy entropy [11]	12	0.956	0.925	0.812	0.971	0.890	–	–	0.927
	30	0.967	0.952	0.852	0.973	0.914	–	–	0.953
	60	0.985	0.968	0.878	0.987	0.935	–	–	0.968

*AUC* area under the curve, *CoSen* coefficient of sample entropy, *NLR* negative likelihood ratio, *NPV* negative-predictive value, *PLR* positive likelihood ratio, *PPV* positive-predictive value, *SVM* support vector machine

^a^AF images were annotated with the non-strict criteria

## Discussion

In this study, we examined the effect of segment window length on the performance of heartbeat interval LP-based AF detection. We used machine learning with CNN for generating discriminant models with varying segment window length. In general, the differences in LP images between non-AF and AF become clearer as the segment window length becomes longer (Fig. 2). Therefore, we originally intended to search for the minimum segment window length required for properly detecting AF. In the training data consisting of LP images consisting either of pure AF or pure SR, the longer the segment window length, the higher the accuracy of AF classification by the CNN models, as expected (Table 3). In the test data consisting of PAF and non-AF, however, the positive likelihood ratio of AF detection showed a convex curve with a peak at around 100 beats. This indicates that, in PAF detection using LP images, if the segment window length of the heartbeat interval is too long, the classification performance rather declines. As the result of precise analysis, the positive likelihood ratio for detecting PAF with 32 × 32-pixel LP image was estimated to peak at a segment window length of 85 beats.

Although the LP of R–R intervals has long been recognized as a useful tool to discriminate AF from other cardiac rhythms [15–18], the present study is the first to report the effects of segment window length on the performance of LP-based AF detection. Anan et al. [15] used LP with R–R intervals of 60-min segments to estimate the functional refractory period of atrioventricular conduction during AF. In a study of the circadian variation of atrioventricular conduction properties during AF, Hayano et al. [11] used LP with 512 beats. In a previous study, we selected LP images with a segment window length of 600 beats for AF detection by CNN [23]. None of these studies, however, have reported the rationale for choosing those segment window lengths for LP. In the present study, we searched for optimal segment length for LP-based PAF detection between 10 and 500 beats. The relationship between PAF detection performance and segment window length varied between performance metrics, but clinically useful metrics (positive likelihood ratio and estimated AF burden) were found to peak at a segment window length of 85 beats.

In this study, we used asymmetric datasets for training and test data for the machine learning. The training data consisted of *pure* LP images containing only AF beats or only SR beats containing few premature beats, while the test data consisted of *mixed* LP images containing various percentages of AF beats from 0 to 100% and also containing premature beats, simulating general clinical situations. This method allowed us to avoid annotating mixed LP images as AF or non-AF by using operational criteria, when generating the training data. Such operational criteria if used may result in generating discriminant models that fit the criteria, and so may the observed optimal segment window length. Instead, we observed how the discriminant models generated using *pure* LP images behave on the mixed LP images. First, we observed that in order for a mixed LP image to be detected as an AF image with 95% probability, it must contain 33 to 79% of AF beats and that the required AF beat ratio was lower for LP images with short (10 beats) and long (500 beats) segment window lengths than for those with intermediate (20 to 200 beats) segment lengths (Table 4). This seems consistent with lower positive-predictive values for the short and long segment window length, suggesting an excessive (or false positive) sensitivity to LP images containing few AF beat (Fig. 3). Second, the observations of the behavior against LP images containing premature beats revealed a progressive decline in AF detection accuracy with increasing premature beat proportion in LP images (Table 5). For any premature beat proportion, however, the accuracy was highest when the segment window length was 100 beats, suggesting the highest robustness against premature beats is obtained at this segment window length. Finally, we observed that the CNN models behaved similarly between CAF and PAF and almost perfectly detected AF when LP images contain only AF beats (Table 6), suggesting the validity of using CAF data to generate CNN models to detect continuous AF beats during PAF episodes.

In this study, we employed the likelihood ratio to determine the optimal segment window length for LP AF detection. The reason for using the likelihood ratio was that while other measures of performance, such as sensitivity, specificity, predictive values, and accuracy, are influenced by the prior probability of events, the likelihood ratio is known to be robust to the probability [24]. In the present study, the LP images were classified as AF if the segments included PAF even one beat. Therefore, the probability of an LP image being annotated as AF increased with the length of segment. Actually, the ratio of LP images classified as AF in the test data was higher for 500-beat LP images (16.1%) than 10-beat LP images (14.5%) (Table 2). With the coarse analysis, we found that the optimal segment window length of LP images for detecting PAF is around 100 beats. Further analysis around 100 beats with fine steps, however, revealed that the positive likelihood ratio showed a variation near the peak (Fig. 4). Since the observed performance metric was considered as a product from the probabilistic sample obtained by random segmentation of the RR intervals with the respective segment lengths, we thought that the position of the true peak should be estimated from the distribution of the data. We therefore performed a regression analysis of the relationships and the peak was estimated to exist at the segment window length of 85 beats.

Several mechanisms may be considered for the finding of this study. The mechanism by which the lower limit of segment window length exists may be straightforward. Assuming that AF is a semi-random sequence of R–R intervals, a certain number of points are required stochastically for the LP to form a characteristic shape to AF. Actually, the confusion matrices for the training data showed that longer segment window lengths improved classification performance (Table 3). In contrast, the mechanisms by which the upper limit of segment window length exists may be more complicated. First, it is obvious that this was caused by the fact that the test data consisted of mixed LP images containing AF, sinus rhythm, and premature beats in a varying proportion. Actually, when all LP images of AF consist only of AF beats, we observed no substantial difference in the detection sensitivity of the LP image between CAF and PAF (Table 6). We also observed that the AF detection accuracy declined with increasing proportion of premature beats in LP images (Table 5). This relationship was observed for all segment window length, but the accuracy also declined with increasing segment window length above 100 beats for any premature beat proportions > 0%. Second, although the proportion of AF beats that need for LP images to be detected as AF was lower for 500-beat LP images than 100-beat images (Table 4), the absolute number of AF beats increased with segment window length (for example, to be detected with 95% provability, 39 and 165 beats for 100- and 500-beat LP image, respectively). Since the median length (IOR) of individual PAF episode was 7 (4–23) beat in the test data, the proportion of mixed LP images consisted of both SR and AF beats increased with the segment window length (Table 2), which may have led a degradation of the AF detection performance when the segment window length exceeded 100 beats. From the above, the existence of the optimal point may be explained as a result of a trade-off between two different segment window length effects that change in the opposite direction each other for the classification performance.

The purpose of the present study was to determine the optimal segment window length for PAF detection by heartbeat interval LP. To avoid the operational definition for annotating mixed LP images containing both SR and AF beats from affecting the observed optimal segment length, we dared to use asymmetric datasets for the training and test data. Nevertheless, the generated CNN model showed an excellent classification performance between non-AF and AF segments. The comparison of the performance using common dataset from the PhysioNet database showed that the classification performance of our models was comparable to or slightly better than those reported for the sophisticated metrics derived from the feature of beat interval time series [9, 11, 13]. Additionally, the optimal segment window length was 85 beats even for the PhysioNet database.

### Clinical implications

We observed that the CNN model of 100-beat LP can detect PAF with 95% probability when 65% of the segment window was occupied by AF beats. This suggests that the onset of PAF episodes can be detected by this model with a delay of 65 beats (⁓ 40 s when the mean heartbeat interval during AF is assumed as 600 ms). Similarly, the end of PAF episodes can be detected with a delay of 35 beats, thus both delays are expected to be offset, resulting in a more accurate measurement of the estimated PAF duration. Actually, we observed that the ratio of LP images of AF agreed with the AF burden of the PAF cases in the test data, with upper and lower limits of agreement of − 0.02 and 0.03, respectively. These support the potential clinical usefulness of this model not only for detecting PAF, but also for estimating the AF burden.

### Limitations

This study has the following limitations. First, although the CAF segments in the training data and the PAF segments included transient atrial flutter or atrial flutter–fibrillation, we included patients with pure atrial flutter in neither training nor test data. Consequently, the results of the present study cannot be extended to the classification between SR and atrial flutter. Similarly, we excluded patients with implanted pacemaker from the study subjects. Our findings are not applicable to such cases. Second, we used asymmetric datasets for the training and test data, i.e., CAF for the training data and PAF for the test data. Thus, swapping the training and test data may give different results, but we did not examine it due to the reason we discussed above. It may be expected, however, that the inclusion of mixed LP images in the training data would lead an improvement of classification performance for the mixed LP images defined by the same annotation criteria. Third, we used fivefold cross-validation to generate discriminant models by CNN. If the goal were to pursue the best discriminatory power, it might be worth looking into other fold numbers. Finally, the accuracy of the measurement of heartbeat interval may influence the performance of AF detection by LP images [13]. In this study, however, we used LP images with a resolution of 32 × 32 pixels, resulting in a pixel size of 80 × 80 ms. Thus, the beat interval measurement accuracy had a tolerance of 10 times the ECG sampling interval (8 ms). Although we also performed AF detection with 64 × 64-pixel LP images, the optimal segment window length was the same (data not shown), but the tolerance of beat interval measurement was halved for that. Given the computational power of currently commercialized Holter ECG analyzers, increasing the resolution of LP images may not be worth the computation time and load.

## Conclusions

Using the machine learning of CNN discriminant models, we examined the effect of segment window length on the performance of PAF detection based on heartbeat interval LP image. We found that the segment window length is an important determinant of the performance and that the optimal segment window length for detecting PAF with 32 × 32-pixel LP image is 85 beats.

## Methods

### Data selections

Study data were obtained from the ALLSTAR database of 24-h ambulatory ECG [20, 21] and the PhysioNet database [22]. All Holter ECG data in the ALLSTAR database were sampled with Holter ECG recorders (Cardy pico series, Suzuken Co., Ltd., Nagoya, Japan) at 125 Hz and 10 bit (0.02 mV/digit) and analyzed by Holter ECG scanners (Cardy Analyzer 05, Suzuken Co., Ltd, Nagoya, Japan). The reliability of the analyzer has been certified by the product conformance test (IEC60601-2-47, International Electrotechnical Commission, Geneva, Switzerland) including assessment of QRS detection accuracy with the American Heart Association (AHA) and Massachusetts Institute of Technology (MIT) ECG databases (the test results are not published to avoid business risks). The ALLSTAR database was used for extracting both the training and test data. The use of this database for this study has been approved by the Institutional Review Board of Nagoya City University Graduate School of Medical Sciences and Nagoya City University Hospital (No. 709). The PhysioNet database was used only for the test data for the comparisons with earlier studies [9, 11, 13].

#### Training data

As the sources of the training data, we extracted 24-h Holter ECG data in 58 subjects with persistent SR and 52 subjects with CAF from ALLSTAR database. The inclusion criterion for the SR data was a 24-h ECG consisting of SR beats > 99% of total beats without PAF episodes or frequent premature beats in subjects > 40 years old. The inclusion criterion for the CAF data was 24-h ECG consisting of persistent AF > 99% of total beats in patients > 40 years old. The data from patients with pacemaker implant were excluded from both the SR and CAF data. The cardiac rhythms were diagnosed and confirmed independently by multiple laboratory technicians and cardiologists. The training data were used for the machine learning to develop CNN discriminant models.

#### Test data

As the sources of the test data, we extracted 24-h Holter ECG data of other 52 subjects with SR without PAF and of 53 subjects with PAF from ALLSTAR database, independently of the training data. In this study, PAF was defined as an AF episode that started and/or ended within each 24-h dataset. The inclusion criterion for SR data was a 24-h ECG consisting of persistent SR beats without PAF episodes in subjects > 40 years old. The inclusion criterion for PAF data was 24-h ECG including at least one PAF episode in patients > 40 years old. For both SR and PAF test data, subjects were selected regardless of the number of premature beats, but patients with pacemaker implant was excluded from both. The cardiac rhythms were diagnosed and confirmed independently by multiple ECG technicians and cardiologists. The onset and offset points of each episode of PAF were determined by agreement between the ECG technicians and confirmed by clinical laboratory technicians. The authors in this research were not involved in these ECG assessments. The test data were used for evaluating the performance of discriminant model obtained from the training data.

Additionally, we used MIT-BIH AF database [6], MIT-BIH Arrhythmia Database [25], and MIT-BIH normal sinus rhythm (NSR) database [22] in the PhysioNet database. These data were used as another test data to compare the performance of the discrimination models generated by CNN with the performance of other methods reported by earlier studies [9, 11, 13].

### Lorenz plot image

We used 24-h time-series data of R–R interval and QRS wave annotations. For the initial coarse analysis, 24-h R–R interval data were split into consecutive non-overlapping segment windows with lengths of 10, 20, 50, 100, 200, and 500 beats for generating LP images. For the secondary detained analysis, the finer steps from 10 beats to one beat were used for the segment window lengths between 50 and 130 beats.

**Fig. 6:**
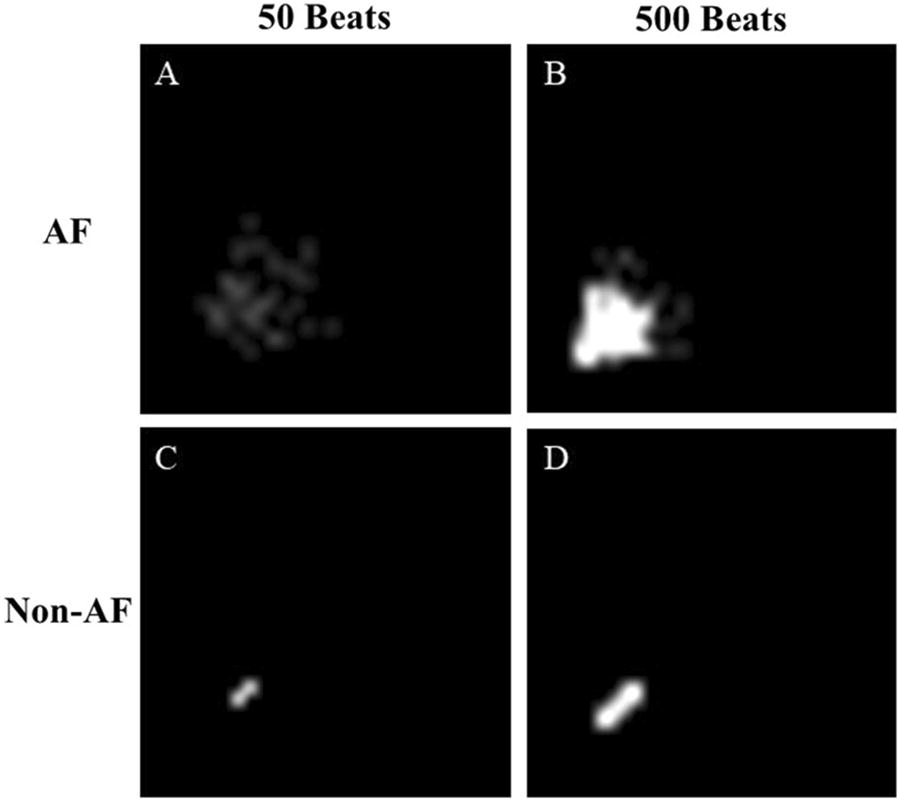
Representative LP images with a 32 × 32-pixel resolution and a 3-bit brightness level for AF (**a**, **b**) and non-AF (**c**, **d**) segments with lengths of 50 (**a**, **c**) and 500 (**b**, **d**) beats. The brightness level reflects the number of points in each pixel

LP was generated for each segment window length by plotting all R–R intervals in the segment but the first one as the *y* values against preceding R–R intervals as the *x* values [15, 17]. The obtained LPs were converted into the monochrome images of a 32 × 32-pixel resolution with 3-bit scale for brightness level, resulting in a temporary resolution of 80 ms and a dynamic range between 0 and 2560 ms for both the *x* and *y* values. When (*x*, *y*) values scaled out of the range, the data were plotted at the edge of the image. The number of plots in each pixel was counted and was used as the brightness level of the pixel. When the number of plots was > 7, the level was set at 7. Figure 6 shows the example of LP images.

### Machine learning with training data

Since the training data were generated from either pure (> 99%) SR or pure (> 99%) CAF cases, all LP images produced for any segment window lengths were consisted of either pure non-AF beats or pure AF beats. Consequently, LP images were simply annotated as non-AF and AF images accordingly.

The machine learning was performed by a CNN using keras (version 2.2.4) open-source python machine learning library, using the Microsoft Windows 10 operating system on a computer equipped with an 8-core Intel Xeon E3-1275 V3 processor with 32-GB memory. The computer was also equipped with an NVIDIA RTX 2080 Ti graphic board with 27-GB memory.

The CNN consisted of an input layer, 1st convolution layer, 2nd convolution layer, 1st max pooling layer, 3rd convolution layer, 2nd max pooling layer, 1st dropout layer, a flatten layer, 1st dense layer, 2nd dropout layer, and 2nd dense layer. In all convolution layers, the kernel size was set at 3 × 3 and the numbers of output filter were set at 32, 64, and 128 in the first, second, and third convolution layers, respectively. In all pooling layers, the height and width were set to be half each. The dropout rate was set at 0.25. In the 1st and 2nd dense layers, the numbers of unit were set at 128 and 2, respectively. Rectified linear unit was used for the activation function of all convolution layers and 1st dense layer and softmax for the activation function of the 2nd dense layer in order to classify LP images. Binary cross-entropy was used for loss function and stochastic gradient descent (SGD) for optimizer. The hyperparameters of SGD were as follows: the learning rate = 0.001, the learning rate decay = 0.000001, and the momentum accelerating SGD = 0.9.

We enrolled fivefold cross-validation for the CNN. Briefly, for each segment window length, the LP images of training data were divided randomly into five subsets. For each cross-validation, one subset out of five was selected in order and used as a validation subset, and the remaining 4 subsets were used as training subsets. Consequently, we obtained five datasets with different training subsets and validation subsets. We set the batch size to 32. Accuracy and loss were updated for each batch. The number of the maximum epochs was set at 50 times per training. If the validation loss did not improve during 10 epochs, training stopped early. As a result, we obtained five models for each segment window length. The validation accuracy and the validation loss of each dataset were updated for each epoch. We obtained the final validation accuracy and the final validation loss of each dataset in the last epoch. Classification accuracy was evaluated by a cross-validation score and the confusion matrices. For the cross-validation score, probabilities were calculated by applying each of the five discriminant models to each LP image in the corresponding data subset from which the model was derived. LP images were classified as AF when the probability was ≥ 0.5 and as non-AF when the value < 0.5. The cross-validation score was obtained as the average accuracy of the five models.

### Classification of test data

In contrast to the training data, the test data were generated from either non-AF or PAF cases regardless of the number of premature beats. Thus, the produced LP images could contain AF beats at various ratios between 0 and 100%. We annotated them with strict and non-strict criteria. In the strict criteria, LP images were annotated as AF images if they contained AF beat even one beat and LP images were annotated as non-AF images if they contained no AF beat at all. In the non-strict criteria, LP images were annotated as AF image if they contained AF beats > 1/2 of total beats in the segment, and non-AF images otherwise. The strict criteria were used for the test data from the ALLSTAR database to examine the optimal segment window length for PAF detection. The non-strict criteria were used for the test data from both ALLSTAR and PhysioNet databases to compare the classification performance between the models developed in the present study and those reported by earlier studies [9, 11, 13].

The confusion matrices for the test data were obtained as follows. For test data of each segment window length, five discriminant models were applied to all of each LP images in the test data and five probabilities were calculated. LP images were classified as AF when the average of the five probabilities was ≥ 0.5 and as non-AF when it was < 0.5. The classification results were summarized as a confusion matrix for each segment window length.

To examine if AF burden is estimated by LP-based AF detection, AF burden was calculated as the ratio of AF beats among the total recorded beats in each 24-h dataset and compared with the ratio of AF LP images among the total LP images generated from each 24-h dataset for each segment window length.

### Statistical analysis

The receiver operating characteristics curve and the area under the curve were calculated for the classification performance in the test data. The sensitivity, specificity, positive-predictive value, negative-predictive value, accuracy, and positive and negative likelihood ratios of classifications were calculated for each segment window length. The relationships between these performance metrics and segment window length were analyzed by regression curve fitting. The logarithmic transformation of axis was used as necessary to the better fitting of regression curves. Among these indices of classification performance, the optimal segment window length was determined based on the likelihood ratios that are known to provide a fair evaluation for classification performance independent of prior probability [24]. To examine if AF burden is estimated by LP-based AF detection, AF burden was calculated as the ratio of AF beats among the total recorded beats in each 24-h dataset and compared with the ratio of AF LP images among the total LP images generated from each 24-h dataset for each segment window length. The agreement between the AF burden and the ratio of AF LP images was evaluated with the upper and lower limits of agreement of Bland and Altman method [26]. These analyses were performed with numpy (version 1.15.4) and scikit-learn (version 0.20.2) open-source python machine learning library.

## Data Availability

The data used and/or analyzed during the current study are available from the corresponding author on reasonable request.

## Abbreviations

AF: Atrial fibrillation
ALLSTAR: Allostatic State Mapping by Ambulatory ECG Repository
CAF: Chronic atrial fibrillation
CNN: Convolutional neural network
LP: Lorenz plot
PAF: Paroxysmal atrial fibrillation
SGD: Stochastic gradient descent
SR: Sinus rhythm

## References

[1] Wolf PA, Abbott RD, Kannel WB (1991). Atrial fibrillation as an independent risk factor for stroke: the Framingham Study. Stroke.

[2] Kimura K, Kazui S, Minematsu K, Yamaguchi T (2004). Japan Multicenter Stroke Investigators C: hospital-based prospective registration of acute ischemic stroke and transient ischemic attack in Japan. J Stroke Cerebrovasc Dis.

[3] January CT, Wann LS, Alpert JS, Calkins H, Cigarroa JE, Cleveland JC, Conti JB, Ellinor PT, Ezekowitz MD, Field ME (2014). 2014 AHA/ACC/HRS guideline for the management of patients with atrial fibrillation: executive summary: a report of the American College of Cardiology/American Heart Association Task Force on practice guidelines and the Heart Rhythm Society. Circulation.

[4] Gallagher MM, Camm AJ (1997). Classification of atrial fibrillation. Pacing Clin Electrophysiol.

[5] Go AS, Reynolds K, Yang J, Gupta N, Lenane J, Sung SH, Harrison TN, Liu TI, Solomon MD (2018). Association of burden of atrial fibrillation with risk of ischemic stroke in adults with paroxysmal atrial fibrillation: the KP-RHYTHM Study. JAMA Cardiol.

[6] Moody GB, Mark RG (1983). A new method for detecting atrial fibrillation using R–R intervals. Comput Cardiol.

[7] Tateno K, Glass L (2001). Automatic detection of atrial fibrillation using the coefficient of variation and density histograms of RR and deltaRR intervals. Med Biol Eng Comput.

[8] Dash S, Chon KH, Lu S, Raeder EA (2009). Automatic real time detection of atrial fibrillation. Ann Biomed Eng.

[9] Lian J, Wang L, Muessig D (2011). A simple method to detect atrial fibrillation using RR intervals. Am J Cardiol.

[10] Zhou X, Ding H, Ung B, Pickwell-MacPherson E, Zhang Y (2014). Automatic online detection of atrial fibrillation based on symbolic dynamics and Shannon entropy. Biomed Eng Online.

[11] Liu C, Oster J, Reinertsen E, Li Q, Zhao L, Nemati S, Clifford GD (2018). A comparison of entropy approaches for AF discrimination. Physiol Meas.

[12] Lin YZ, Yu SN (2018). Bispectrum and histogram features for the identification of atrial fibrillation based on electrocardiogram. Conf Proc IEEE Eng Med Biol Soc.

[13] Oster J, Clifford GD (2015). Impact of the presence of noise on RR interval-based atrial fibrillation detection. J Electrocardiol.

[14] Lorenz EN (1963). Deterministic nonperiodic flow. J Atmos Sci.

[15] Anan T, Sunagawa K, Araki H, Nakamura M (1990). Arrhythmia analysis by successive RR plotting. J Electrocardiol.

[16] Garcia-Alberola A, Yli-Mayry S, Block M, Haverkamp W, Martinez-Rubio A, Kottkamp H, Breithardt G, Borggrefe M (1996). RR interval variability in irregular monomorphic ventricular tachycardia and atrial fibrillation. Circulation.

[17] Hayano J, Sakata S, Okada A, Mukai S, Fujinami T (1998). Circadian rhythms of atrioventricular conduction properties in chronic atrial fibrillation with and without heart failure. J Am Coll Cardiol.

[18] Mizutani M (1989). Analysis of the RR interval in patients with atrial fibrillation using a Lorenz-Plot method. Rinsho Byori.

[19] Sharifi S, Pakdel A, Ebrahimi M, Reecy JM, Fazeli Farsani S, Ebrahimie E (2018). Integration of machine learning and meta-analysis identifies the transcriptomic bio-signature of mastitis disease in cattle. PLoS ONE.

[20] Hayano J, Kisohara M, Yoshida Y, Sakano H, Yuda E (2019). Association of heart rate variability with regional difference in senility death ratio: ALLSTAR big data analysis. SAGE Open Med.

[21] Hayano J, Kisohara M, Ueda N, Yuda E (2020). Impact of heart rate fragmentation on the assessment of heart rate variability. Appl Sci.

[22] Goldberger AL, Amaral LA, Glass L, Hausdorff JM, Ivanov PC, Mark RG, Mietus JE, Moody GB, Peng CK, Stanley HE (2000). PhysioBank, PhysioToolkit, and PhysioNet: components of a new research resource for complex physiologic signals. Circulation.

[23] Kisohara M, Masuda M, Yuda E, Hayano J: Neural Network Detection of Atrial Fibrillation by Lorenz Plot Images of Interbeat Interval Variation. In: 2018 IEEE 7th Global Conference on Consumer Electronics (GCCE); Nara, Japan. IEEE; 2018.

[24] Flemons WW, Littner MR (2003). Measuring agreement between diagnostic devices. Chest.

[25] Moody GB, Mark RG (2001). The impact of the MIT-BIH arrhythmia database. IEEE Eng Med Biol Mag.

[26] Bland JM, Altman DG (1986). Statistical methods for assessing agreement between two methods of clinical measurement. Lancet.

